# Supplementary material for: A new taekwondo-specific field test for estimating aerobic power, anaerobic fitness, and agility performance
Source: PLoS One. 2022 Mar 16;17(3):e0264910. doi: 10.1371/journal.pone.0264910 (PMC8926267; doi:10.1371/journal.pone.0264910)
Supplement: Attachment — Submitted filename: PONE_Reviewer Report_20211119.docx [file pone.0264910.s005.docx]

**Reviewer Comments**

**A new taekwondo-specific field test for estimation aerobic power, anaerobic fitness, and agility performance**

This study has the originality of creating a Taekwondo-specific fitness test.

**[Introduction]**

Ln81: “...evaluate aerobic and aerobic power...”

\# Are both 'aerobic' correct?

**[Methods]**

Ln115: “different weight categories”

\# You presented no detailed explanation of the “different weight categories.” Why did you use this expression?

Ln124: “being aged 18 to 35 years”

\# In this study, the age range of study subjects was wide. I recommend that you describe the age distribution of the study subjects. This is because there is a significant correlation between fitness and age.

Ln219: “bandal tchagui”

\# “bandal tchagui” → “bandal chagi”

**[Results]**

\# In this study, you tried to predict aerobic power, anaerobic fitness, and agility using the TAAA test. So you developed the VO2max prediction equation. However, you did not provide a predictive equation for anaerobic fitness or agility performance. I think that your presentation of both prediction equations mentioned above makes the results of this study clear.

**[Discussion]**

Ln405-408: “Likewise, the classificatory table provided here can help coaches to monitor their athletes’ progress and classify their performance for specific goals. The 30-sec WAnT constitutes the most common non-specific method used to assess anaerobic power and capacity of taekwondo athletes, and is widely accepted as a valid anaerobic test.”

\# The classificatory table you presented in this study is helpful, and it would also be beneficial to give a regression model to predict the WAnT test result.

Ln415: “bandal tchagui”

\# “bandal tchagui” → “bandal chagi”

\# In general, when developing a new test method, developers divide the collected data by 7:3 or 8:2 and then create a test method using 70-80% of the data. And the feasibility of the developed method is checked using the remaining 20-30% of data. However, this study did not use this validation method. Would you please explain why?
