# Supplementary material for: A new taekwondo-specific field test for estimating aerobic power, anaerobic fitness, and agility performance
Source: PLoS One. 2022 Mar 16;17(3):e0264910. doi: 10.1371/journal.pone.0264910 (PMC8926267; doi:10.1371/journal.pone.0264910)
Supplement: Attachment — Submitted filename: Response to Reviewer 1.docx [file pone.0264910.s007.docx]

**Dear Reviewer**

We are so thankful for your accurate comments in order to reduce existing errors. We have applied your comment in the article, which can be found in yellow color.

Authors must distinguish between 'practical methods' and 'correct methods.' Are the classificatory tables developed by the authors more accurate than Wingate test results? Or could the classificatory table created by the authors replace the Wingate test results? The result of an estimate always has an error. That is why developers should try to reduce this error.
I expect this method created by the authors to be practically used in the Taekwondo field. So, to further reduce the error of this classification table, I proposed the estimation formulas. However, I felt that the authors disagreed with my suggestion that the findings of this study should be made more valid. Therefore, I can't entirely agree with the publication of this paper because I expect that the error of the results of this study will be significant.

**Response:** The regression model was applied to provide the equation that accurately describes the relationships between peak and mean power in the Wingate test and the results of the TAAA test, i.e.:

“The regression models were therefore used to describe the relationship between the correlated variables. The KS test was applied to detect the normality assumption of residuals for maximum (KS = 0.23, *p* < 0.001) and average (KS = 0.09, *p* = 0.2) values. Because this assumption was not met for peak power, the regression model for this variable was not accepted. However, a linear regression model was developed to estimate average power values according to the relationship between average power output obtained from WAnT and average kicks in the TAAA test (t = 8.81; *p* < 0.001), in which the equation that best described this relationship was as follows:

Equation (3)

**Average Power (W.kg^-1^)** = 0.648 (average kicks)

The abovementioned equation could explain 63% of the overall variability between the variables with a SEE of 0.53 W.kg^-1^. The mean and SD of average power for the TAAA test and WAnT were 7.3 ± 0.85 and 7.3 ± 0.68 W.kg^-1^, respectively. The mean CV was 11.7% and 9.3% for the TAAA test and WAnT, respectively. We then performed the ANOVA Levene´s F test to confirm there was no difference in the CV between the tests (F = 2.34; *p* = 0.12).”

**Kind regards,**
